# Selective microdochectomy after ductoscopic wire marking in women with pathological nipple discharge

**DOI:** 10.1186/1471-2407-9-151

**Published:** 2009-05-17

**Authors:** M Hahn, T Fehm, EF Solomayer, KC Siegmann, AS Hengstmann, D Wallwiener, R Ohlinger

**Affiliations:** 1 Department of Obstetrics and Gynaecology, University Hospital Tuebingen, Tuebingen, Germany; 2 Department of Diagnostic and Interventional Radiology; University Hospital Tuebingen, Tuebingen, Germany; 3 Clinic for Trauma, Orthopaedic and Reconstructive Surgery, Catholic Hospital, Hagen, Germany; 4 University Women's Hospital, Greifswald, Germany

## Abstract

**Background:**

To investigate the diagnostic reliability of selective microdochectomy after direct ductoscopic wire marking of suspect lesions in patients with pathological nipple discharge.

**Methods:**

Selective microdochectomy due to pathological discharge was performed in 33 patients with mean age of 51.7 years. Ductoscopes of 0.9 and 1.1 mm in diameter with a channel for wire marking were used. Only patients without sonographic or mammographic correlation for the discharge were included. The pathologic mammary duct was wire marked and extirpated under direct visual guidance via the ductoscope. The histological results were compared with cytology, galactography and ductoscopy.

**Results:**

In 24 out of 33 cases (72%) an intraductal, epithelial proliferation was found histologically. The following sensitivities for intraductal, epithelial proliferations could be determined: cytology 4%, galactography 74%, and ductoscopy 78%.

**Conclusion:**

The method allows selective microdochectomy of the pathological duct and the intraductal proliferation under visual guidance. The resection volume can be reduced in contrast to the unselective ductectomy after injection of methylene blue.

## Background

**Figure 1:**
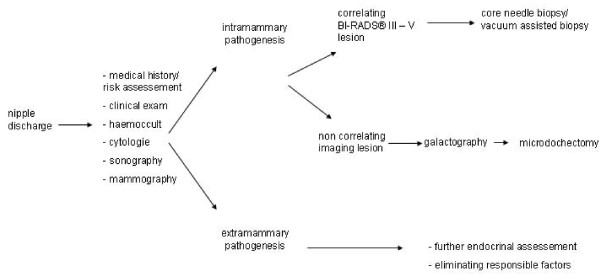
**Diagnostic algorithm for pathological nipple discharge**.

Pathological nipple discharge is the only symptom which cannot yet be histologically clarified by minimal invasive procedures such as core needle biopsy or vacuum assisted biopsy in clinical routine. It is define as spontaneous, persistent, unilateral and coming from a single duct during non-lactational period [1]. The imaging techniques mammography and galactography [2,3] as well as sonography and magnetic resonance imaging (MRI) cannot replace histological examination in patients with pathologic nipple discharge (Fig. 1). Controversy exists in the diagnostic value of nipple discharge cytology [4-6]. In 10 to 15% of the cases, pathological discharge is the only symptom of breastcancer [7-10]. Techniques like major duct excision and microdochectomy are used for histological clarification [1,11-13]. Here, a coloured liquid dye (methylene blue) is instilled into the affected lactiferous duct, making the duct visible to the surgeon. Finally, the coloured duct is dissected through an infraareolar incision and excised with the surrounding tissue in a cone shape. Since lactiferous ducts divide into bifurcations, it is understandable that this procedure might be nonselective. This is because the instilled dye may disperse into a duct system which is not responsible for the discharge. Ductoscopy has the advantage of direct visualisation of the intraductal lesion [14-21].

The aim of this prospective study was to investigate the diagnostic reliability of selective microdochectomy after direct ductoscopic wire marking of pathologic lesions in patients with pathological nipple discharge and to clarify whether the method represents an alternative to standard procedure using methylene blue dye.

## Methods

### Patients

33 consecutive women who presented with pathologic nipple discharge between April 2007 and September 2008 were included in this prospective study. The study was conducted under the guidelines of the local ethics committee, and in accordance with the principles of the Declaration of Helsinki. The mean age of the patients was 51.7 years (range 20 – 71 years). The inclusion criteria were: pathologic nipple discharge due to breast pathology coming from only one lactiferous duct, imaging without abnormal findings that could be biopsied and consent of the patients to take part in the study. No patient had to be excluded from the study and all underwent complete workup with nipple smear, mammography, sonography, and galactography.

A selective microdochectomy was performed after ductoscopic wire marking. An origin for the discharge outside the breast was excluded in all women, and we proceeded according to the algorithm shown in Fig. 1. Women were only included in the study if the discharge appeared unilaterally from only one lactiferous duct. A cytological nipple smear was taken from 32 women. One patient declined the nipple smear. Alongside the clinical examination, all women received a mammography (Senographe 2000D, GE, Fairfield) galactography (Ultravist 300, Schering, Berlin) as well as breast ultrasound examination (IU 22, 12 MHz, Philips, Hamburg). Mammography and sonography were classified according to BI-RADS^®^. The BI-RADS^® ^classification (breast imaging reporting and data system) of the ACR (American College of Radiology) provides a standardised classification of imaging findings according to the likelihood of malignancy. Patients who had a mammographic or sonographic origin for the discharge as well as a palpable lesion were excluded from the study and biopsied using a minimal invasive technique.

The study was perfomed in cooperation with the Working Group for Minimal Invasive Breast Interventions of the German Senology Society.

### Microdochectomy/Ductoscopy

**Figure 2:**
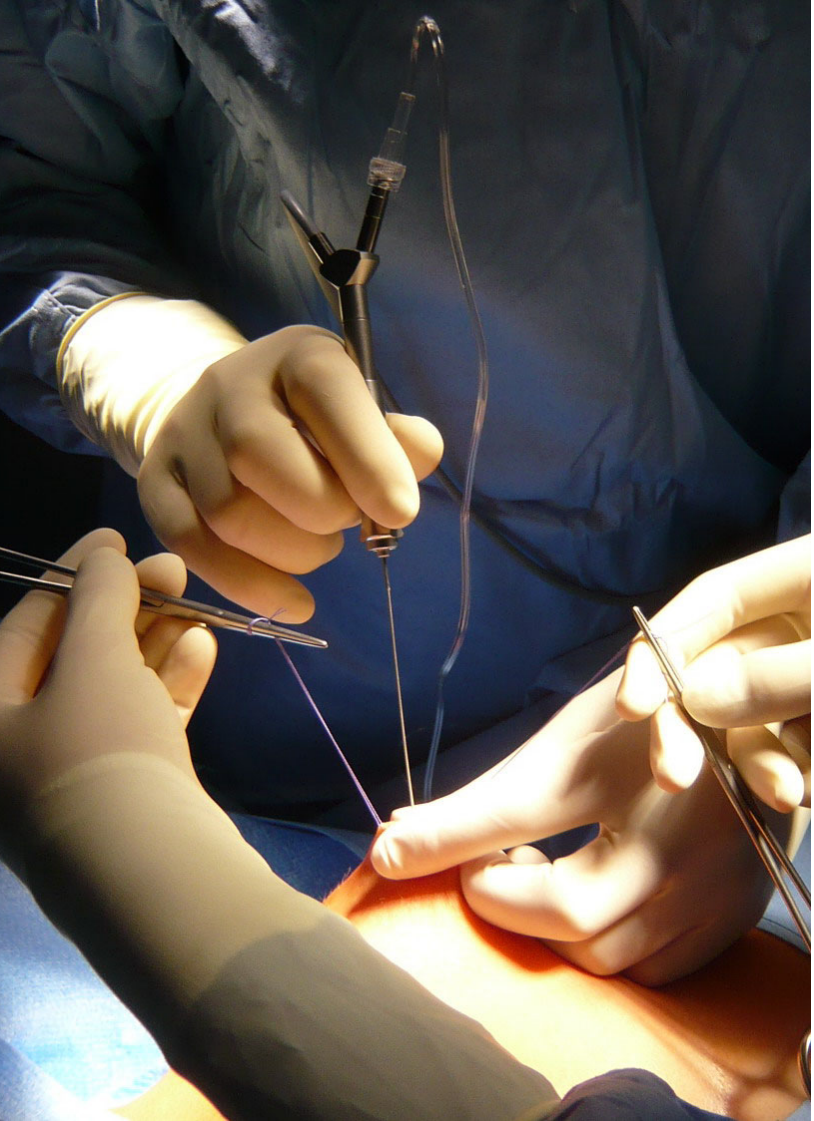
**Insertion of the ductoscope**.

**Figure 3:**
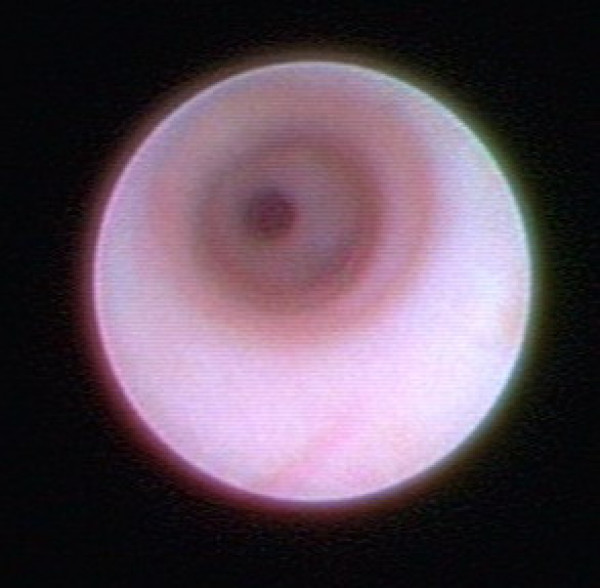
**Normal lactiferous duct**.

**Figure 4:**
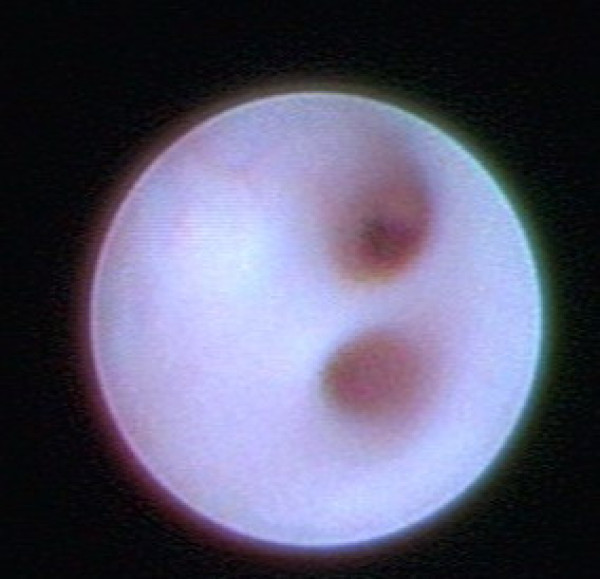
**Pignose sign (bifurcation)**.

**Figure 5:**
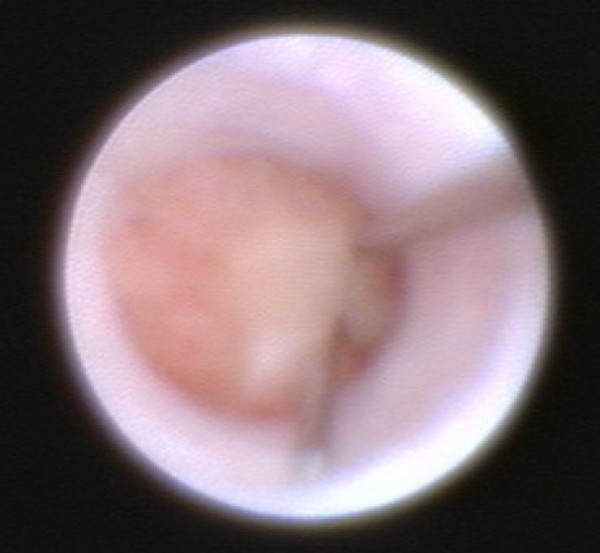
**Wire marking of the intraductal papilloma**.

**Figure 6:**
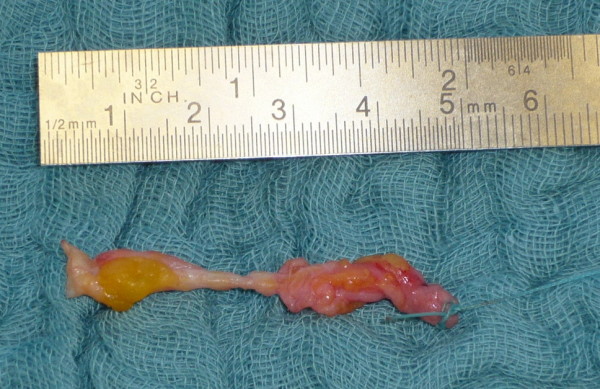
**Selective lactiferous duct sample**.

All procedures were performed under general anaesthesia. The mean duration of the procedure was 32 minutes (Range: 30–50 minutes). After preoperative marking of the planned infraareolar incision, the discharging lactiferous duct was dilated using Hegar's dilators to 1.2 mm. Finally, the ductoscope (Karl Storz, Tuttlingen, 1.1 mm diameter with working channel, 0.9 mm without working channel, semiflexible) was introduced into the duct (Fig. 2). The ductoscope possesses two working channels. Channel 1 allows the continuous supply of sodium chloride 0.9% solution for dilatation of the mammary ducts. Channel 2 allows insertion of the wire marker. An endocamera and conventional monitor screen were used for image visualisation. The lesions were documented photographically. After passage of the proximal section of the milk duct (Fig. 3) and arrival at the first bifurcation (Pignose sign) (Fig. 4), the surgeon is challenged with which milk duct is to be further examined. In most cases, this can be solved with the so-called „jet-stream" sign, a backflow of the pathological secretion, which can be triggered by pressure on the dorsal section of the breast. The backflow of the pathological discharge indicates the correct duct to follow. If this was not possible, all following mammary ducts were explored, as far as technically possible. The detected lesion was finally marked with a wire (Somatex, Teltow) through the working channel (Fig. 5). A reliable correlation between image and histology is therefore comprehensible. When the wire marker was positioned, the ductoscope was removed from the breast. Surgical microdochectomy followed using hydrodissection, after subcutaneous injection of 20–30 ml prilocaine 1% with epinephrine 1:200000. After the wire-marked milk duct was dissected, it was suture marked retroareolarly for orientation (Fig. 6). Haemostasis and glandular adaptation with wound closure finally followed in the usual manner.

Patients received a thoracic compression bandage for 24 hours and were asked to return for follow-up one week postoperatively.

## Results

### Ductoscopy

The ductoscope could be introduced into the correct lactiferous duct in all 33 cases. A mean of 4 lactiferous ducts were ductoscopically examined during each procedure (range 1–12). Bloody discharge was present in 29 cases, and a serous discharge in 4 cases. Flat intraductal red deposits were diagnosed ductoscopically in 6 cases, polypoid structures in 18 cases, and in 9 cases no abnormality was found (including 3 cases of false passage). In the case of a false passage, surgery was performed via a retroareolar cone extirpation according to Urban. In one case, the false passage already arose during galactography and the perforation site could be detected ductoscopically. In all 3 cases of false passage, a papilloma was detected histologically.

The detected intraductal lesions or the proximal section of the pathological lactiferous ducts could be ductoscopically marked in all cases.

In order to gain a representative tissue sample, at least a further 2 cm of the duct, distally to the suspect lesion seen via ductoscopy, was also included in the resection.

We realized a learning curve of about 20 procedures.

### Galactography

32 galactographies were performed. One patient declined the procedure. Infiltration of the milk duct with contrast media was inadequate in 5 cases. An intraductal mass was diagnosed galactographically in 20 cases, while in 7 cases the lactiferous ducts were galactographically unsuspicious.

### Histology and Cytology

Twenty-two papillomas, 1 invasive ductal carcinoma with intraductal component (DCIS), 1 DCIS, 1 galactophoritis, 1 lymphocytic lobulitis as well as 7 other benign results were diagnosed histologically.

The cytological smears were negative (foamy macrophages, rare single cells, clear background secretion) in 29 cases, papillomatous cells were diagnosed in 2 cases and in 2 cases an analysis was not possible. In the 2 cases where cytology revealed papillomatous cells, this result correlated in one case with the histology, and in the second case, a lymphocytic lobulitis was found.

Table 1 shows the results of galactography, ductoscopy and cytology compared to histological results. The highest detection rate could be determined by ductoscopy, followed by galactography.

**Table 1:** Detection rate of ductoscopy, galactography and cytology in comparison to histological results (n = 32, galactography was refused in one case)

	Intraductal, epithelial proliferations N = 23	No intraductal proliferations N = 9
**Ductoscopy**		
positive	18 (78%)	5 (56%)

negative	2 (9%)	4 (44%)

False passage	3 (13%)	0

**Galactography**		
positive	17 (74%)	3(33%)

negative	2 (9%)	5 (56%)

not analysable	4 (17%)	1 (11%)

**Cytology**		
positive	1 (4%)	1 (11%)

negative	20 (87%)	8 (89%)

not analysable	2 (9%)	0

### Complications

Medical complications such as haematomas requiring revision and secondary bleeding, inflammation requiring antibiotics or objectionable cosmetic results did not occur. In one case where the lactiferous duct had to be dissected very close to the areola, there was a temporary reduction in blood perfusion with partial necrosis. This healed well with conservative treatment.

In 2 cases, there was breakage of the fibreoptic fibres in the ductoscope, and the procedure had to be continued with a replacement ductoscope. We believe that the ductoscope broke because the surgeon tried to enter a milk duct which was at too steep an angle. The flexibility of the used ductoscopes are limited.

### Follow up

The patients were examined one week postoperatively. Patients with benign findings were advised to attend for a clinical examination including mammography and sonography after 6 months.

No conclusion can be made about recurrence due to the short follow-up period.

## Discussion

The clarification of pathological nipple discharge is still a particular diagnostic challenge [9]. On the one hand, this is because the imaging techniques such as mammography and sonography do not have a high diagnostic value when it comes to pathologic nipple discharge; on the other hand, this symptom cannot yet be histologically confirmed with minimal invasive techniques.

In this study intraductal epithelial proliferation could be histologically detected in 73% (24/33) of cases. In all of these 24 cases, the lesions could not be diagnosed either by clinical examination, sonography or mammography.

Galactography successfully demonstrated an intraductal lesion that could be successfully confirmed histologically in 73% (17/23) of the cases. In 33% (3/9 cases) a false positive galactography occured (histological confirmation could not be made on the postoperative specimen). We recommended a 6 month follow up for these patients (sonography).

Ductoskopy successfully demonstrated an intraductal lesion that could be histologically confirmed in 78% (18/23). Two false negative results occured from ductocopy. In both cases papillomas could be histologically diagnosed. The invasive ductal carcinomas with intraductal component as well as the DCIS were both detected by ductoscopy (Table 1).

In those 3 cases of galactography that were false positive the ductoscopy showed a flat intraductal lesion in 1 case and no intraductal lesion in 2 cases. In those 5 cases of false positive ductoscopy the galactography showed an intraductal mass in 1 case and in 4 cases an unsuspicious lactiferous duct.

False passage occurred in three cases. This negatively influenced the interpretation of the study because of the small number of cases. It should be pointed out that one false passage already occurred during the galactography and the other two occurred very early in the study. We interpret this as being a result of our steep learning curve.

The challenge in the clarification of patients with pathological nipple discharge in one collective, as described in this study, (i.e. without imaging correlation from mammography or sonography) is the histological confirmation. [14]. Open biopsy according to microdochectomy [1,11,12], i.e. after instillation of methylene blue dye into the pathological duct only allows an indirect view of the lactiferous ducts from the exterior. In combination with galactography [2,3,22-26] it is possible for the surgeon to identify the blue coloured lactiferous duct system and excise them. With this technique however, microdochectomy follows without direct visualisation of the lesion. This means that the resection volume must be relatively large and that the surgeon has no intraoperative control as to whether the intraductal proliferation, if present at all, was removed or not. If the pathologist reports an inconspicuous lactiferous duct, the question of the pathogenesis of the pathological discharge remains unknown. Four differential diagnostic possibilities can be causative here: 1) extirpation of the wrong lactiferous duct, 2) the biopsy was too superficial, i.e., the lesion lies more distal, 3) loss of the lesion during the pathological work up, 4) no intraductal proliferation which is responsible for the pathological discharge exists.

The follow-up in this particular situation is difficult. As a result of dissection of the lactiferous duct, the symptom of bloody discharge should no longer occur, assuming the correct duct has been removed. Clinical examination, sonography and mammography, along with MRI for specific questions, remain the only follow-up investigations. After non-representative ductectomy, intraductal lesions might be recognised sonographically inside a duct ectasia caused by a discharge blockage. However, a control mammography should be performed in these patients, even though the interpretation of the images can be hindered by postoperative scars.

Here the advantage of direct ductoscopic visualisation of the lesion to be removed is evident. Both the surgeon and pathologist gain information as a result of ductoscopic detection and marking of the suspect lesion. The pathologist can also be informed about the depth of the site.

Whether microdochectomy still has to be performed when a lesion has not been detected ductoscopically cannot be answered with the current data. False negative biopsies can also occur under ductoscopic visualisation. One reason for this could be a non-representative ductectomy after dilating a false lactiferous duct. Another reason for false negative biopsies is that the presence of further lesions distal to a discovered intraductal lesion cannot be excluded. This is why the galactography result showing the complete length of the lactiferous duct is important information for the surgeon.

A further advantage of ductoscopic marking is the reduction in the resection volume compared to standard procedure with methylene blue dye. According to our experience, the resection volume under ductoscopic visualisation was subjectively smaller than that of the conventional ductectomy after methylene blue instillation. An objective study of these parameters has, nevertheless, not been carried out in the available studies.

It also should be mentioned that the abdication of methylene blue dye using ductoscopy might be meaningful. Cases have been described in the literature of tissue necrosis after application of methylene blue dye[27,28] which has been associated with a number of local complications due to its tissue reactive properties. Some authors have therefore suggested replacing methylene blue with an alternative dye.

Considering the future prospects of technical equipment development, it would be desirable to develop a ductoscopic minimal invasive method for clarification of pathological breast discharge. The first experiences of minimal invasive clarification of intraductal lesions solely with the ductoscope have been described in the literature [29-31]. Innovative approaches such as ductoscopic-vacuum assisted biopsy removal of intraductal lesions avoiding open biopsy or instruments using the working channel of the ductoscope for cytological or histological samples [32] are already technically realisable today. Similarly, it is also conceivable to biopsy a ductoscopically discovered lesion with classical vacuum assisted breast biopsy under combined sonographic-ductoscopic control. However, these procedures are still in the experimental stages.

## Conclusion

Microdochectomy after ductoscopic wire marking allows specific histological clarification of intraductal lesions under direct visualisation in the case of pathological nipple discharge. A reduction in the resection volume compared to the standard ductectomy after methylene blue dye administration appears to be possible with this technique.

## Competing interests

The authors declare that they have no competing interests.

## Authors' contributions

MH performed the study conception. MH and RO participated in the study design. MH performed the data acquisition. MH, ES and AH participated in the data analysis. TF, ES, KS, AH, DW and RO carried out the critical revision of manuscript. All authors read and approved the final manuscript.

## Pre-publication history

The pre-publication history for this paper can be accessed here:

http://www.biomedcentral.com/1471-2407/9/151/prepub
